# Supplementary material for: Accelerating Molecular Dynamics through Informed Resetting
Source: J Chem Theory Comput. 2025 Jan 8;21(2):605–13. doi: 10.1021/acs.jctc.4c01238 (PMC11781593; doi:10.1021/acs.jctc.4c01238)
Supplement: Supplementary file 1 — ct4c01238_si_001.pdf [file ct4c01238_si_001.pdf]

Supporting Information: Accelerating Molecular Dynamics through Informed Resetting

Jonathan R. Church,[†,‡] Ofir Blumer,[†,‡] Tommer D. Keidar,[‡] Leo Ploutno,[‡] Shlomi Reuveni,[¶,‡,§] and Barak Hirshberg[*,¶,‡,§]

†*J.R.C. and O.B. contributed equally to this work*

‡*School of Chemistry, Tel Aviv University, Tel Aviv 6997801, Israel*

¶*The Center for Computational Molecular and Materials Science, Tel Aviv University, Tel Aviv 6997801, Israel*

§*The Center for Physics and Chemistry of Living Systems, Tel Aviv University, Tel Aviv 6997801, Israel*

E-mail: hirshb@tauex.tau.ac.il

The Mean Number of Resetting Events

We analyzed the mean number of restarting events for the simulations of the modified Faradjian-Elber potential of Figure 1A of the main text. The results are shown in Figure S1. We found that the number of average resetting events for each threshold correlated with the qualitative behavior of the speedup curves given in the main text. For thresholds which exhibited a plateau at large resetting rates we found that there was a monotonous increase in the number of average resetting events with the resetting rate until reaching a plateau. On the other hand, for thresholds in which the speedup increased, reached a

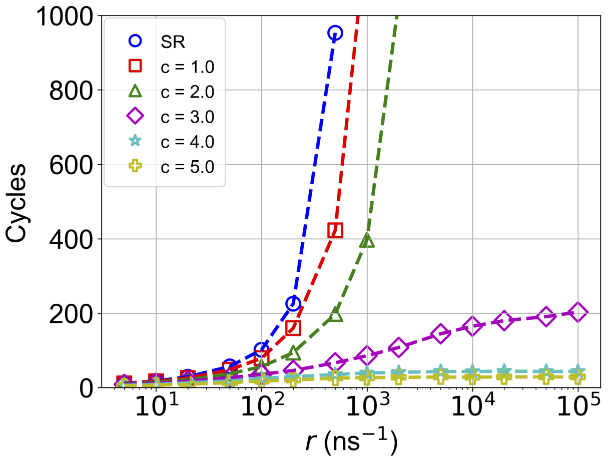

Figure S1: The mean number of resetting events when using ISR and SR for resetting rates ranging from 5 to 10^5 ns^{-1}.

maximum value, and then decreased we found that the mean number of resetting events continued to increase as the resetting rate was increased.

The Effect of the Initial Position

We analyzed the effect of restarting from different initial positions on the resulting speedup using the Modified Faradjian-Elber potential. The speedups obtained are presented in Figure S2. Here, the initial position of the particle was taken to be $x = 2, 4$ Å and $y = 0$ Å (Figures S2A, and S2B, respectively.)

We found the same qualitative behavior as in the main text for all initial positions that started with $y = 0$ Å, with the exception that the threshold leading to the greatest acceleration changed depending on the restarting location, as expected.

Symmetric Double-well Potential

We used the symmetric double-well potential to test if ISR can accelerate systems which do not exhibit any speedup when using standard SR (see Figure S3). The equation for the

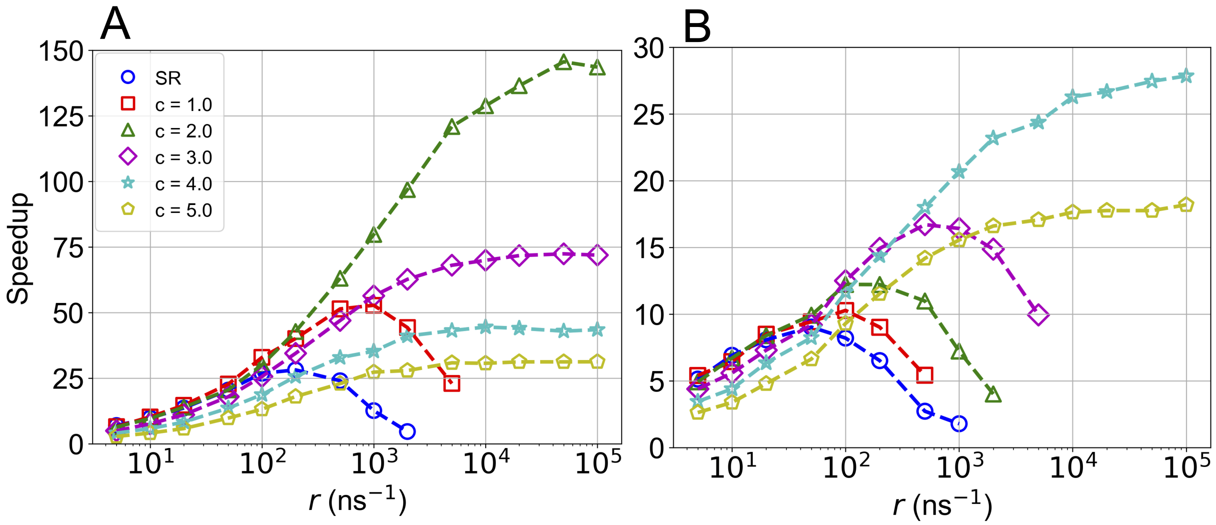

Figure S2: Speedups obtained when changing the starting location of the particle using standard SR and ISR: A) $(x, y) = (2,\ 0)$ Å, and B) $(x, y) = (4,\ 0)$ Å. The acceleration obtained for ISR and SR were studied for resetting rates ranging from 5 to 10^5 ns^{-1}.

symmetric double-well potential is given by Equation S1,

$$V(x, y) = A_1 x^4 - A_2 x^2. \tag{S1}$$

Here, we modified the values of A_1 and A_2 in order to achieve the desired position of the minima and barrier height. For the potential with minima located at ±2.5 Å with a 1 k_BT barrier A_1 and A_2 were 9.404×10^{17} Jm^{-4} and 1.176×10^{-1} Jm^{-2}, respectively. For the elongated potential where the minima were positioned at ±5 Å with a 2 k_BT barrier, A_1 and A_2 were 1.176×10^{17} Jm^{-4} and 5.878×10^{-2} Jm^{-2}, respectively. The trajectories were initiated from the positive x minimum, x = 2.5 Å or 5 Å, and propagated until reaching the negative x minimum, -2.5 Å or -5.0 Å. For the potential in Figure S3A, the MFPT was 28.44 ps, while raising the barrier (See Figure S3C) produced a MFPT of 183.60 ps.

This potential was selected because we found empirically that no acceleration could by gained by standard resetting (see "SR" in Figures S3B and S3D). By using ISR (Figures S3B and S3D), however, we were able to obtain accelerations of up to $\sim$ 50%. Similar to the results obtained using the Modified Faradjian-Elber Potential, the optimal threshold was

associated with the initial position of the particle.

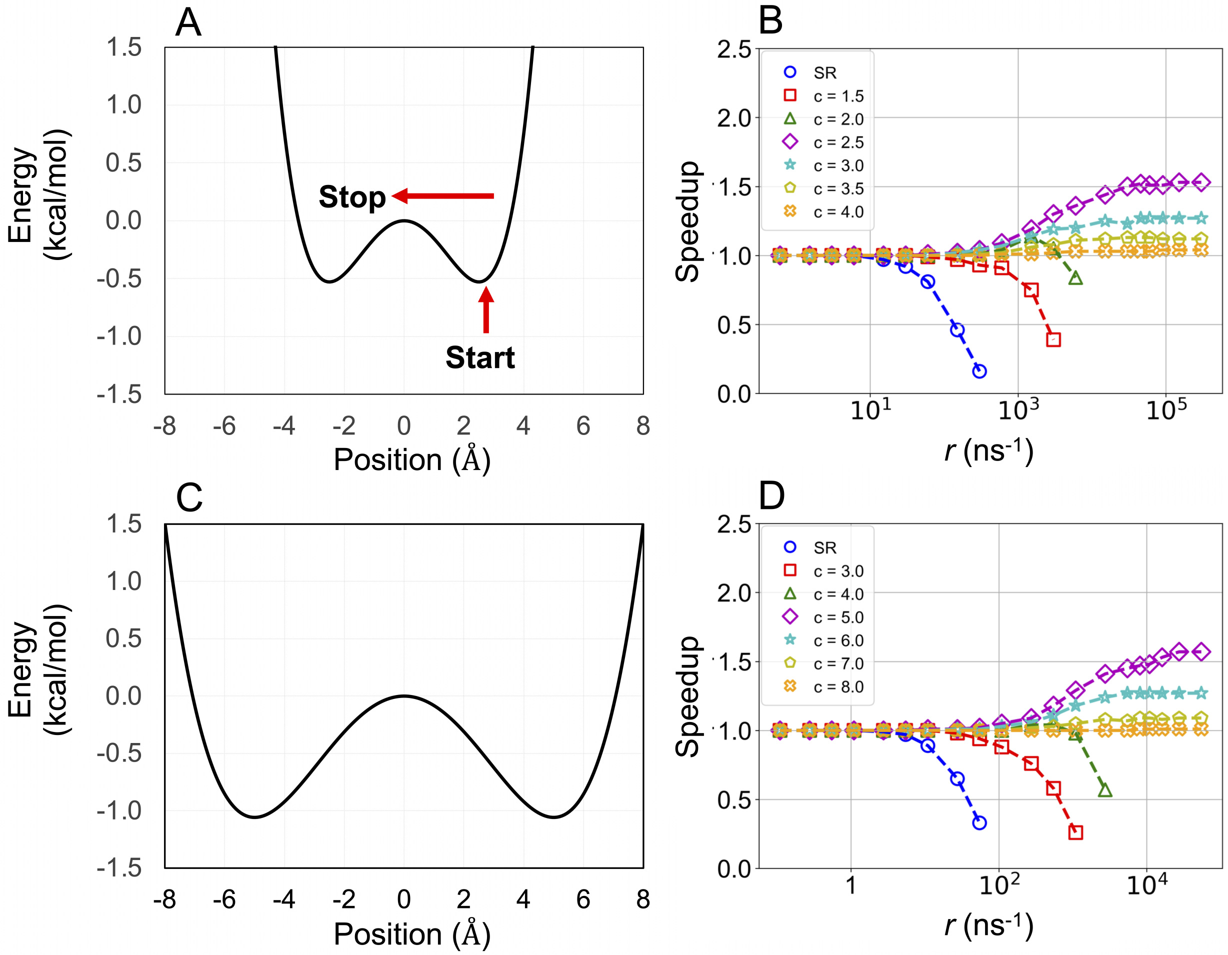

Figure S3: A) Symmetric Double Well potential with a 1.0 $k_B T$ barrier and minima at $\pm$ 2.5 Å, B) the speedups obtained from using SR and ISR, C) Symmetric Double Well potential with a 2.0 $k_B T$ barrier and minima at $\pm$ 5 Å, D) the speedups obtained from the elongated double well potential.

Committor analysis

We conducted a committor analysis to validate our assumption that the x coordinate is the optimal CV for the modified Faradjian-Elber potential. We followed the procedure described by Peters.[S1] We first ran a 10 ns long simulation initiated at the origin, trapped around $x = 0$ Å using a trapping potential $V(x) = 500x^2$, with V being in units of $1\,k_B T$ and x in Å. Then, we sampled 1000 configurations along the biased trajectory. We initialized 100 trajectories

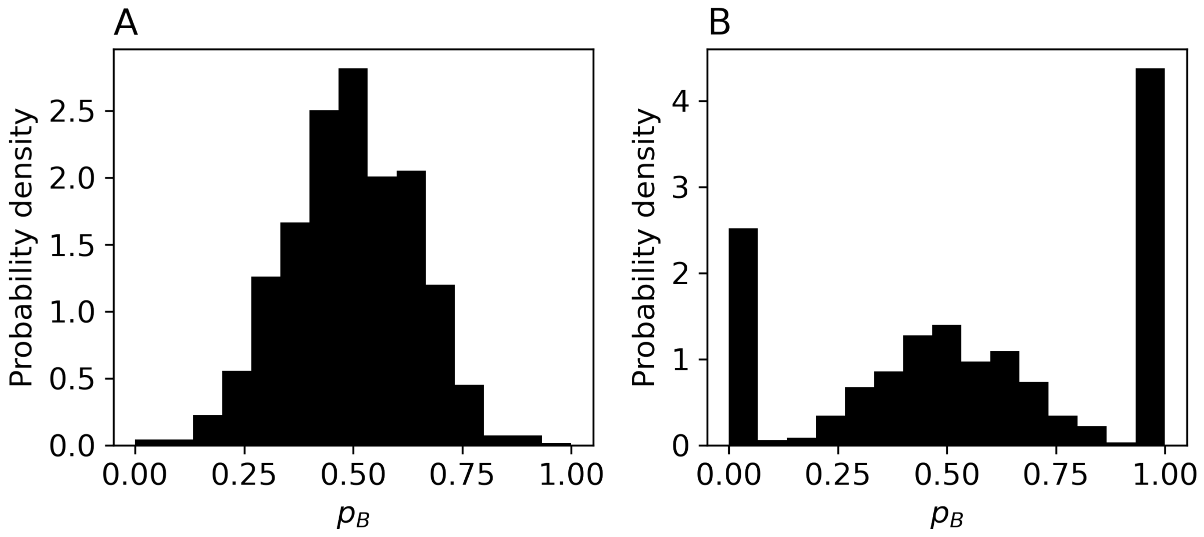

Figure S4: Histograms of p_B for the modified Faradjian-Elber potential, for A) the x coordinate or B) a CV rotated 3° relative to the x-axis.

from each configuration. The trajectories where terminated when reaching either minima $x = 3$ Å or $x = -3$ Å, denoted states A and B, respectively. Finally, we obtained p_B, the fraction of trajectories reaching state B before reaching A, for each configuration, and plot the histogram of p_B in Figure S4A. The histogram is symmetric, with a peak at $p_B = 0.5$, as expected for the committor. For comparison, we performed the same procedure for a CV rotated by 3° relative to the x-axis. In this case, we sampled initial configurations from two 10 ns simulations initiated at the origin. We used the same trapping potential, but also rotated by 3° relative to the x-axis. The histogram, given in Figure S4B, has two peaks, at $p_B \to 0$ and $p_B \to 1$, due to an overlap between the basins and the transition state along the CV, confirming it is a suboptimal one.

Proof that no additional simulations are required for $r > r*$

Here, we prove that the ensemble of trajectories with ISR and resetting rate $r^* + \Delta r$ is statistically equivalent to the ensemble of trajectories obtained by performing ISR at rate $\Delta r > 0$, on trajectories with ISR at rate r^*. First, note that the resetting strategy is

fully characterized by Equation 3 in the main text. So by showing that the probability of resetting is the same for two strategies, we prove that the ensembles of trajectories created by using them are equivalent. As a result, no additional simulations are required to predict the ISR results at rate $r^* + \Delta r$ for the kinetics inference. We use the standard prediction procedure, only treating the trajectories with resetting at a rate r^* as the underlying process and predicting their behavior under a resetting rate Δr.

Consider first a process undergoing ISR at rate r^*. According to Equation 3 in the main text, the probability of undergoing resetting is

$$p_{r^*}(\boldsymbol{X}) = \begin{cases} r^*\Delta t & \text{if } \boldsymbol{X} > c, \\ 0 & \text{otherwise.} \end{cases} \tag{S2}$$

Adding ISR at rate Δr on top, resetting at positions $\boldsymbol{X} > c$ could happen via two independent resetting mechanisms. The original one, which occurs with probability $r^*\Delta t$ and the newly added one with probability $\Delta r \Delta t$. The total probability for resetting is their sum

$$p_{r^*,\Delta r}(\boldsymbol{X}) = \begin{cases} r^*\Delta t + \Delta r \Delta t & \text{if } \boldsymbol{X} > c, \\ 0 & \text{otherwise,} \end{cases} \tag{S3}$$

which is just the probability, $p_{r^*+\Delta r}(\boldsymbol{X})$, of resetting the original process at an ISR rate of $r^* + \Delta r$, which concludes the proof.

References

(S1) Peters, B. In *Reaction Rate Theory and Rare Events Simulations*; Peters, B., Ed.; Elsevier: Amsterdam, 2017; pp 539–571.
